# Comparative Study of Antioxidant Status in Androgenic Embryos of *Aesculus hippocastanum* and *Aesculus flava*

**DOI:** 10.1155/2014/767392

**Published:** 2014-02-03

**Authors:** Dubravka Štajner, Boris M. Popović, Dušica Ćalić, Marijana Štajner

**Affiliations:** ^1^Faculty of Agriculture, University of Novi Sad, Trg Dositeja Obradovića 8, 21 000 Novi Sad, Serbia; ^2^Department of Plant Physiology, Institute for Biological Research “Siniša Stanković,” University of Belgrade, Despota Stefana Boulevard 142, 11000 Belgrade, Serbia; ^3^Emergency Centre, Clinical Centre of Vojvodina, Hajduk Veljkova 1, 21000 Novi Sad, Serbia

## Abstract

*In vivo* (leaves and seed embryos) and *in vitro* (androgenic embryos) antioxidant scavenging activity of *Aesculus hippocastanum* and *Aesculus flava* medical plants was examined. Here we report antioxidant enzyme activities of superoxide dismutase, catalase, guaiacol peroxidase and glutathione peroxidase, reduced glutathione quantity, flavonoids, soluble protein contents, quantities of malondialdehyde, and ^•^OH radical presence in the investigated plant samples. Total antioxidant capacity of all the samples of *A. hippocastanum* and *A. flava* was determined using FRAP, DPPH, and NO^•^ radical scavenger capacity. The leaves of *A. flava* collected from the botanical garden exhibited stronger antioxidant activity (higher activities of SOD, and higher quantities of GSH, TSH, TPC, and scavenging abilities of DPPH and NO^•^, and higher FRAP values and lowest quantities of ^•^OH and MDA) than *in vitro* obtained cultures. However, the leaves of *A. flava* showed higher antioxidant activity than the leaves of *A. hippocastanum*, and therefore they have a stronger tolerance of oxidative stress. Androgenic embryos of both species had low amount of antioxidants due to controlled *in vitro* environmental conditions (T, photoperiod, humidity, nutritive factors, and pathogen-free). Our results confirmed that we found optimal *in vitro* conditions for producing androgenic embryos of both *Aesculus* species. Also, we assume that horse chestnut androgenic embryos can be used as an alternative source for large-scale aescin production.

## 1. Introduction

Horse chestnut (*Aesculus hippocastanum *L.) grows under varying ecological conditions in many European cities in the northern temperate zone [1, 2]. Yellow buckeye (*A. flava *Marshall) is a species of buckeye native to Florida, USA.* A. flava* as well as many American *Aesculus *species is resistant to the *C. ohridella* leaf miner. *A. hippocastanum* and *A. flava* have a slow and difficult reproduction cycle under natural conditions, which can be overcome via *in vitro* androgenesis.

*Aesculus* species have different medicinal or cosmetic uses, and the bark of the horse chestnut contains low amounts of gallic and tannic acids which are used in industrial applications. The bark and leaves of *A. hippocastanum* have been employed as an astringent to treat diarrhea and hemorrhoids, venous insufficiency, and postoperative edema in order to pass kidney stones and to ease stomach aches, while a fraction of the seed was swallowed to alleviate hemorrhoidal symptoms [3]. *A. hippocastanum* increases the antioxidative defense system of the body and prevents HFD-induced lipid peroxidation in male mice [4]. In mainland China, the seeds of *A. chinensis* have been used as stomachic and analgesic in the treatment of distention and pain in the chest and abdomen and in the treatment of malaria and dysentery and heart diseases [5].

Saponins from *A. hippocastanum* have been reported to show anti-inflammatory activity [6]. It was proven that Japanese horse chestnut (*Aesculus turbinata* Blume) suppresses the blood glucose levels using the oral starch tolerance test and long-term antiobesity effects in obese mice fed a high-fat diet. Recently, it was reported that seed shells of *A. turbinata* contain higher levels of polyphenolic antioxidants than typical foods such as cranberry, blueberry, almonds, hazelnut, and chestnut [7–9]. The antioxidant compounds can be recycled in the cell or are irreversibly damaged, but their oxidation products are less harmful or can be further converted to harmless substances [10, 11].

Plant *in vitro* cultures are able to produce and accumulate many medicinally valuable secondary metabolites [12–17]. Many different *in vitro* approaches have been used for increased biosynthesis and for the accumulation of antioxidant compounds in plant cells. *In vitro* technology offers some or all of the following benefits: simpler extraction and purification from interfering matrices, novel products not found in nature, independence of climatic factors and seasons, more control over biosynthetic routes for obtaining the most desired variants compounds, shorter and more flexible production cycles, and easier fulfillment of the high-profile pharmaceutical production [18]. Biotechnological methods based on *in vitro* tissues and plants are considered as raw material for producing standardized material, independent from environmental factors [19–22]. The presence of substantial amounts of aescin in androgenic embryos of *A. hippocastanum*, which remained high after a few years of culture and could be increased further by applying certain plant growth regulators, was detected [23].

In the present paper, we evaluated the antioxidant capacities of extracts obtained from leaves and zygotic embryos *in vivo *and androgenic embryos *in vitro* of *A. hippocastanum* and* A. flava*. Antioxidant activities of the extracts from *in vitro* cultures were compared with those of extracts of *A. hippocastanum* and *A. flava* grown in nature. The aim of this research was to study the antioxidant scavenging activity in globular and cotyledonary androgenic embryos of *A. hippocastanum* and* A. flava *with the goal of improving the experimental *in vitro* culture growth conditions.

## 2. Material and Methods

### 2.1. Plant Material

Leaves, seed embryos (as control), and anthers were collected from elite *A. hippocastanum* and *A. flava *trees. *A. hippocastanum* and *A. flava* were harvested during April. Inflorescences with closed flower buds were transported and stored in the dark at 4°C.

Completely closed flower buds (4-5 mm long) were used in the experiments. The selected buds were surface sterilized with 95% ethanol and 70% ethanol for about 5 min, followed by three rinses in sterile distilled water. Basal medium contained [24] mineral salts MS, and 2% sucrose and was supplemented with the following (mg L^−1^): pantothenic acid 10, nicotinic acid 5, vitamin B_1_ 2, adenine sulphate 2, myo-inositol 100, and casein hydrolysate 200 and 0.7% agar. Induction MSS medium contained basal medium enriched with 2,4-D dichlorophenoxyacetic acid (2,4-D) and kinetin (KIN), 1.0 mg L^−1^ of each. Six or seven anthers were inoculated in each culture tube containing 8 mL of the induction medium. Embryo development and multiplication of androgenic embryos from anther culture proceeded on MS medium with reduced concentration of 2,4-D (0.01 mg L^−1^) and the same concentration of KIN after 60 days. After which, the medium for the multiplication of embryos was cultured on MS hormone-free medium for embryo maturation.

All media were sterilized by autoclaving at 0.9 × 10^5^ KPa and 114°C for 25 min. Cultures were grown at a temperature of 25 ± 1°C and a 16 h photoperiod with irradiance of 33–45 *μ*mol m^−2^ s^−1^ produced by cool white fluorescent tubes. Plant material used in the experiment is presented in Table 1.

**Figure 1:**
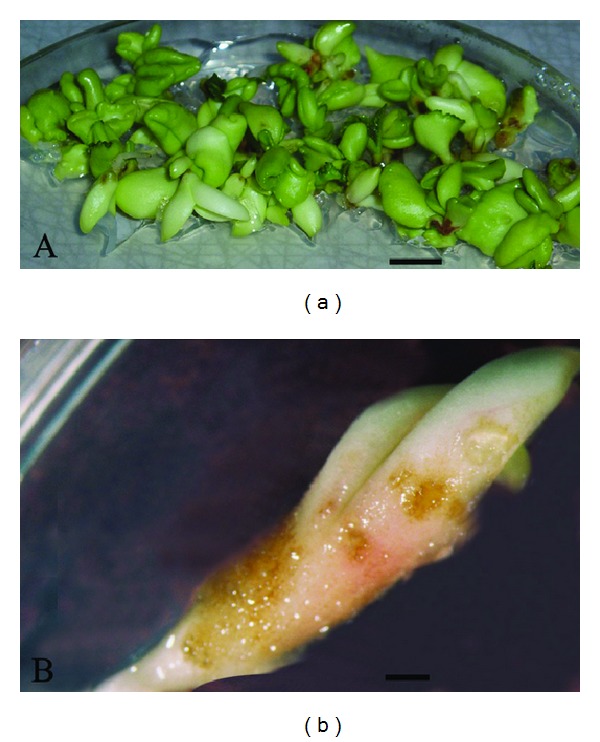
Androgenic embryos in cotyledonary stage of *Aesculus hippocastanum* (a) and *Aesculus flava* (b) cultivated on MS hormone-free medium. Scale bar: (a) 10 mm; (b) 1 mm.

Androgenic embryos showed rapid differentiation and asynchronous development. Globular, heart-like, torpedo and cotyledonary embryos appeared after 8 weeks of androgenesis induction for both species. However, androgenic embryos of both species in the early (globular embryos) and late (cotyledonary embryos, Figure 1) stages of development were used in experiments.

### 2.2. Extraction Procedures

Plant material (1 g) was extracted with 25 mL 70% aqueous ethanol (0.1 M HCl) and sonicated for 30 min in an ultrasonic bath at ambient temperature. The extracts were rapidly vacuum-filtered through a sintered glass funnel and kept refrigerated. This extract was used for total phenolic content, DPPH and NO^•^ radical scavenger capacity (RSC), and total antioxidant power determinations.

For lipid peroxidation, antioxidant enzymes, hydroxyl radical quantity and soluble protein content, and phosphate buffer (pH 7) extracts were used. One gram of plant material was extracted with 50 mL 0.1 M K_2_HPO_4_ at pH 7.0 after 30 min of sonication in an ultrasonic bath at ambient temperature. After 10-minute centrifugation at 4°C and 15 000 g, the aliquots of the supernatant were used for the above-mentioned determinations.

### 2.3. Total Phenol Content

Total phenol content (TPC) was determined spectrophotometrically using the Folin and Ciocalteu assay described by [25]. Aliquots of plant extracts (250 *μ*L) were mixed with 4.0 mL distilled water and 250 *μ*L of previously diluted Folin and Ciocalteau reagent. Aliquots of saturated Na_2_CO_3_ solution (500 *μ*L) were added to this mixture to produce basic conditions. The mixture was diluted to 10 mL with distilled water. The absorbance versus a prepared blank was read at 760 nm until it reached steady state. The same procedure was applied for six standard solutions of catechin (50–300 mg/100 mL). Final results were expressed as mg catechin equivalent per 100 g dry sample.

### 2.4. Total Antioxidant Capacity

#### 2.4.1. FRAP

Total antioxidant capacity was estimated according to the FRAP (Ferric reducing antioxidant power) assay [26]. The FRAP reagent was prepared by mixing: acetate buffer (300 mM pH 3.6), TPTZ (2,4,6-tripyridyl-s-triazine) reagent (10 mM in 40 mM HCl), and FeCl_3_·6H_2_O (20 mM) in ratio 3 : 1 : 1. Sample (100 *μ*L) was mixed with 3 mL of working FRAP reagent and absorbance (593 nm) was measured at 4 minutes after vortexing. FRAP value was calculated using the formula:

(1) $$\begin{array}{c} \text{FRAP value}=\frac{\Delta A_{\text{sample}}\left(0\text{–}4\;\min\right)}{\Delta A_{\text{standard}}\left(0\text{–}4\;\min\right)}, \end{array}$$

FRAP unit is equal to 100 *μ*M Fe^2+^/dm^3^ Fe^2+^.

#### 2.4.2. DPPH^•^ Radical Scavenging Capacity

DPPH^•^ RSC assay was based on measurement of the loss of DPPH (2,2-diphenyl-1-picrylhydrazyl) color after reaction with test compounds [27]. The DPPH^•^ radical is one of the few stable organic nitrogen radicals, which bears a deep purple color. This assay is based on the measurement of the reducing ability of antioxidants toward DPPH^•^. The ability can be evaluated by measuring the decrease of its absorbance. The widely used decoloration assay was first reported by [28]. Each extract (5, 10, 20, 30, and 40 *μ*L) was mixed with 90 *μ*M DPPH^•^ in methanol making up a final volume of 3.0 mL. The mixtures were shaken vigorously and were stored in dark for 30 min at room temperature. The decrease of absorbance of the reaction mixtures for the control was monitored spectrophotometrically at 515 nm.

RSC was calculated by following:

(2) $$\begin{array}{c} \text{RSC}=\left(\frac{\left(A_{0}-A_{1}\right)}{A_{0}}\right)\cdot 100. \end{array}$$

#### 2.4.3. ^•^NO Radical Scavenging Capacity

^•^NO RSC was evaluated by measuring the accumulation of nitrite (formed by the reaction of NO with oxygen), according to the Griess reaction [29]. NO^•^ was generated by sodium nitroprusside in buffered aqueous solution. Each prepared extract (10, 25, 50, 75, and 100 *μ*L) was mixed with freshly prepared solution of sodium nitroprusside (0.5 mL, 0.01 M in NaH_2_PO_4_-Na_2_HPO_4_ buffer, 0.067 M, pH 7.4) and NaH_2_PO_4_-Na_2_HPO_4_ buffer (0.067 M, pH 7.4) making a final volume of 1.0 mL. These mixtures were prepared at 25°C for 10 min and illuminated at 3000 lx. After illumination, each reaction mixture (1 mL) was mixed with Griess reagent (1 mL, 0.1% N-(1-naphthyl)-ethylenediamine dihydrochloride (NEDA) in distilled water and 1% sulfanilamide in 5% H_3_PO_4_). Reduction of nitrite by the extracts was determined spectrophotometrically at 546 nm, by measuring the decrease of absorbance of the reaction mixtures for the control (containing the same chemicals except for the sample).

RSC was calculated by following:

(3) $$\begin{array}{c} \text{RSC}=\left(\frac{\left(A_{0}-A_{1}\right)}{A_{0}}\right)\cdot 100. \end{array}$$

### 2.5. Lipid Peroxidation

Lipid peroxidation was estimated based on thiobarbituric acid (TBA) reactivity. Samples were evaluated for malondialdehyde (MDA) production using a spectrophotometric assay for TBA. The extinction coefficient at 532 nm of 153,000 mol^−1^ cm^−1^ for the chromophore was used to calculate the MDA-like TBA produced. The colour intensity of the MDA-TBA complex in the supernatant was measured by its absorbance at 532 nm [30].

### 2.6. Antioxidant Enzymes

Enzyme specific activity is expressed as *μ*mol of the substrate transformed in minute/mg protein. Superoxide dismutase (SOD, EC 1.15.1.1.) activity was determined by the method based on the inhibition of adrenaline transformation to adrenochrome at pH 10.2. SOD units can be regarded as the amount of enzyme which causes a 50% inhibition in the extinction change in 1 min as compared to the control [31]. Measurements were made at 480 nm.

Guaiacol peroxidase (GPx, EC 1.11.1.7.) activity was determined using guaiacol as substrate at 436 nm [32]. Glutathione peroxidase (GSH-Px, EC 1.11.1.9.) activity was determined using cumene hydroperoxide and reduced glutathione (GSH) as substrates at 412 nm [33]. Catalase (CAT, EC 1.11.1.6.) activity was determined at 240 nm. The decomposition of H_2_O_2_ was followed by decrease in absorbance [34].

The amount of reduced GSH and total thiols (TSH) was determined with Ellman's reagent at 412 nm [35]. Soluble protein content was determined [36]. Hydroxyl radical (^•^OH) was determined by the inhibition of deoxyribose degradation [37].

### 2.7. Statistical Analysis

For each parameter, experiments and extraction procedures were performed in triplicate. All measurements for each extract were also recorded in triplicate. Statistical comparisons between samples performed with Duncan *t*-test for independent observations were done using STATISTICA 9.1. Differences were considered significant at *P* < 0.05.

## 3. Results and Discussion

### 3.1. TPC and Total Antioxidant Status *In Vitro* and *In Vivo* Tissues of *A. flava*

This is the first report about antioxidant scavenging activity in androgenic embryos of *A. hippocastanum* and* A. flava.* We chose two *Aesculus* species because they are related. *A. flava* is often grafted on *A. hippocastanum* for improving cold and insect resistance [2].

The results obtained from the study are presented in four comparative tables containing data concerning *in vivo *control samples (leaves and seed embryos), *in vitro* globular, and *in vitro* cotyledonary embryos of *A. hippocastanum *(Figure 1(a)) and* A. flava *(Figure 1(b)).

Significant differences in MDA, ^•^OH, FRAP, NO^•^ RSC, DPPH^•^ RSC, and TPC were observed (Table 1) in all investigated samples of *A. flava*. TPC was the highest in leaves of control plant 173.8 (mg gallic acid/100 g) and the lowest in globular *in vitro* embryos 47.7 (mg gallic acid/100 g). Apart from the TPC leaves of the control plant exhibited the highest values of DPPH^•^ RSC (28.9%), NO^•^ RSC (51.4%), and FRAP values (744.3 FRAP units). The lowest scavenging activities of DPPH^•^ (12.7%) and NO^•^ (17.1%) were observed in globular *in vitro* embryos. The highest MDA (33.8 nmol MDA/mg protein) and ^•^OH (62.4 nmol/mg protein) quantities were observed in globular *in vitro* embryos which indicate greater disintegration of membrane lipids [10]. On the other hand, accumulation of the ^•^OH radical was the highest in globular androgenic embryos which agrees with statements of other authors who observed that the O_2_^•−^ generation rate and H_2_O_2_ level, (H_2_O_2_ could be decomposed and generate ^•^OH radicals) [10] increased in tissue culture, respectively, and were higher than in the normal tissue [38]. The lowest MDA (11.7 nmol MDA/mg protein) and ^•^OH (15.4 nmol/mg protein) quantities were observed in leaves of a *A. flava* control plant which is the consequence of high scavenging activities and TPC content. Similar results were obtained by other authors on *Centaurea *L. species [39, 40].

The results presented showed that the investigated samples of *A. flava* were exposed to the negative influence of oxidative stress but also showed that they possess effective antioxidant capacity indicating a possible benefit which may be explored in future.

Comparative data concerning antioxidative enzymes activities reduced glutathione and total thiol content in *A. flava in vivo* and *in vitro* samples are presented in Table 2. SOD (1586.1 U/mg protein) activity was the highest in leaves of *A. flava* control plant, as well as quantities of GSH (3.1 *μ*mol/mg protein) and TSH (4.1 *μ*mol/mg protein). SOD present in leaves removes O_2_^•−^ in the compartments where O_2_^•−^ radicals are formed including chloroplast and mitochondria, controlling oxidative stress in plants [41]. CAT activity was the highest in androgenic embryos in cotyledonary stage (31.6 nmol/mg protein), as well as GPx activity (1515.2 nmol/mg protein). Content of soluble proteins was the highest in globular androgenic embryos 2.1 (mg/g).

The results presented showed that all investigated samples of *A. flava* suffered from the negative consequences of oxidative stress but also showed that they possess effective antioxidant capacity indicating a possible benefit which should be further explored. On the basis of almost all parameters of antioxidant status, we could conclude that leaves of *A. flava *exhibited stronger antioxidant activity (higher activities of SOD, higher quantities of GSH, TSH, TPC, scavenging abilities of DPPH^•^ and NO^•^, higher FRAP values, and lowest quantities of ^•^OH and MDA) than *in vitro* obtained cultures. Our previous results showed that plant leaves possess the highest antioxidant activity comparing to other plants organs [42–44].

### 3.2. TPC and Total Antioxidant Status *In Vitro* and *In Vivo* Tissues of *A. hippocastanum*

Results concerning *A. hippocastanum *are presented in Tables 3 and 4. Results from Table 3 clearly indicated that seed embryos control exhibited the highest antioxidant ability due to the highest TPC content (194.4 mg gallic acid/100 g), scavenging abilities of DPPH^•^ (36.5%), NO^•^ (39.2%), and the highest FRAP values as well (338.6 FRAP units). Similar ratio between DPPH^•^, FRAP, and total phenols was observed in guava [45]. Other authors also stated that the significant relationship between antioxidant capacity and total phenolic content indicates that phenolic compounds are valuable contributors to the antioxidant properties of these plants [46]. On the other hand, ^•^OH (59.2 nmol/mg protein) and LP (29.7 nmol MDA/mg protein) were the lowest in the leaves of a control plant which is supported by previous studies [47].

Results presented in Table 4 indicate that SOD activity was the highest (3197 U/mg protein) as well as quantities of GSH (3.4 *μ*mol/mg protein) and TSH (4.1 *μ*mol/mg protein) which together with the lowest ^•^OH and MDA quantities indicate that their high antioxidative capacity (Tables 3 and 4) in leaves of *A. hippocastanum* was similar as in leaves of *A. flava*. This is in agreement with the finding of previous studies that GSH and TSH are necessary to maintain the normal reduced state of cells and that they are potential scavengers of the most dangerous ^•^OH radical [41].

On the basis of our results for antioxidant power *in vitro A. hippocastanum *samples, we could conclude that both control samples seed embryos and leaves exhibited high antioxidative power because they employ antioxidant defense systems to protect themselves against ROS. If we compared leaves of *A. flava* and *A. hippocastanum, *we observed that leaves of *A. flava *exhibited higher antioxidant ability and therefore a stronger tolerance of oxidative stress.

Researching the resources of plants may bring new and safe natural products into pharmaceutical, cosmetic, and food industries [48]. Research showing that combinations of different natural antioxidants present in medicinal plants work better than separate antioxidants alone [49] has increased interest among scientists towards exploring natural antioxidants from botanical sources and those produced in tissue culture. Our results indicated that extracts of *A. flava* and *A. hippocastanum *control samples and tissue culture materials exhibited antioxidant and scavenging abilities. Our investigation could be the starting point for further phytochemical investigations of *A. flava* and *A. hippocastanum in vitro *plants. Androgenic embryos of *A. hippocastanum* and *A. flava* had low amount of antioxidants due to the controlled environmental conditions we employed (T, photoperiod, humidity, nutritive factors, and pathogen-free). It can be concluded that tissue culture methods produce optimal condition for the growth of *Aesculus *androgenic embryos.

## 4. Conclusions

*In vivo *control samples (leaves) ofboth speciesshowed higher antioxidant activity than *in vitro* obtained androgenic embryos. However, *A. flava *leaves had better antioxidant activity than the leaves of *A. hippocastanum*, and therefore they have a stronger tolerance of oxidative stress.

The optimization of *in vitro* conditions for mass production of androgenic embryos could improve cultivation techniques and achieve diversity protection, conservation of these species, and protection from leaf miner* Cameraria ohridella*.

These results could be also beneficial for growing *Aesculus *plants with a high tolerance to oxidative stress and also for producing a physiology stable standardized material independent of environmental factors.

## Figures and Tables

**Table 1:** Total phenolic content, DPPH and NO RSC, FRAP, ^•^OH quantity, and lipid peroxidation in *A. flava*.

*Aesculus flava* samples	TPC (mg catechin/100 g)	DPPH RSC (%)	NO RSC (%)	FRAP (FRAP units)	^•^OH (nmol/mg protein)	LP (nmol MDA/mg protein)
*In vitro* androgenic embryos in cotyledonary stage	61.23^a^	16.38^a^	28.11^a^	237.0^a^	38.44^a^	18.95^a^
*In vitro* androgenic embryos in globular stage	47.70^b^	12.71^a^	17.11^b^	185.4^b^	62.39^b^	33.83^b^
Leaves *in vivo* (control)	173.8^c^	28.95^b^	51.38^c^	744.3^c^	15.36^c^	11.68^c^

Values in rows marked with different letters (a, b, c, and d) were significantly different according to Duncan *t*-test *P* < 0.05. For each parameter, experiments and measurements were also recorded in triplicate; TPC: total phenol content; RSC: radical scavenging capacity; FRAP: Ferric reducing antioxidant power; LP: lipid peroxidation.

**Table 2:** Soluble protein content, antioxidant enzyme activities (SOD, GPx and CAT), and glutathione, and total thiol content in *A. flava. *

*Aesculus flava* samples	Proteins (mg/g)	SOD (U/mg protein)	GPx (nmol/mg protein)	CAT (nmol/mg protein)	GSH (*µ*mol/mg protein)	TSH (*µ*mol/mg protein)
*In vitro* androgenic embryos in cotyledonary stage	1.0^a^	1024^a^	1515^a^	31.6^a^	2.7^a^	2.7^a^
*In vitro* androgenic embryos in globular stage	2.1^b^	333.8^b^	1493^a^	14.3^b^	1.0^b^	1.0^b^
Leaves *in vivo* (control)	0.9^a^	1586.0^c^	36.9^b^	15.3^b^	4.1^c^	4.1^c^

Values in rows marked with different letters (a, b, c, and d) were significantly different according to Duncan *t*-test *P* < 0.05. For each parameter, experiments and measurements were also recorded in triplicate; SOD: superoxide dismutase; GPx: glutathione peroxidase; CAT: catalase; GSH: reduced glutathione; TSH: total thiols.

**Table 3:** Total phenolic content, DPPH and NO RSC, FRAP, ^•^OH quantity, and lipid peroxidation in *A. hippocastanum *samples.

*Aesculus hippocastanum* samples	TPC (mg gallic acid/100 g)	DPPH RSC (%)	NO RSC (%)	FRAP (FRAP units)	^•^OH (nmol/mg protein)	LP (nmol MDA/mg protein)
*In vitro* androgenic embryos in cotyledonary stage	72.0^b^	14.0^b^	35.7^b^	152.3^b^	11.6^b^	10.4^a^
Leaves *in vivo* (control)	35.6^a^	16.3^b^	25.4^c^	134.6^b^	59.2^c^	29.7^b^
Seeds *in vivo* (control)	194.4^c^	36.5^c^	39.2^b^	338.6^c^	4.5^d^	2.8^c^

Values in rows marked with different letters (a, b, c, and d) were significantly different according to Duncan *t*-test *P* < 0.05. For each parameter, experiments and measurements were also recorded in triplicate; TPC: total phenol content; RSC: radical scavenging capacity; FRAP: ferric reducing antioxidant power; LP: lipid peroxidation.

**Table 4:** Soluble protein content, antioxidant enzyme activities (SOD, GPx and CAT), and glutathione, and total thiol content in *A. hippocastanum*.

*Aesculus hippocastanum* organs	Proteins (mg/g)	SOD (U/mg protein)	GPx (nmol/mg protein)	CAT (nmol/mg protein)	GSH (*µ*mol/mg protein)	TSH (*µ*mol/mg protein)
*In vitro* androgenic embryos in cotyledonary stage	2.2^b^	500.5^b^	1290^b^	11.9^b^	0.9^b^	0.9^b^
Leaves *in vivo* (control)	0.6^c^	3197^c^	55.9^c^	30.9^a^	3.4^c^	4.1^c^
Seeds *in vivo* (control)	8.4^d^	123.8^d^	327.7^d^	47.8^c^	0.7^b^	0.5^b^

Values in rows marked with different letters (a, b, c, and d) were significantly different according to Duncan *t*-test *P* < 0.05. For each parameter, experiments and measurements were also recorded in triplicate; SOD: superoxide dismutase; GPx: glutathione peroxidase; CAT: catalase; GSH: reduced glutathione; TSH: total thiols.
